# High-Gain High-Field Fusion Plasma

**DOI:** 10.1038/srep15790

**Published:** 2015-10-28

**Authors:** Ge Li

**Affiliations:** 1 Institute of Plasma Physics, Chinese Academy of Sciences (ASIPP), PO Box 1126, Hefei, Anhui 230031, PRC.; 2 School of Nuclear Science and Technology, University of Science and Technology of China, Hefei, Anhui 230029, PRC

## Abstract

A Faraday wheel (FW)—an electric generator of constant electrical polarity that produces huge currents—could be implemented in an existing tokamak to study high-gain high-field (HGHF) fusion plasma, such as the Experimental Advanced Superconducting Tokamak (EAST). HGHF plasma can be realized in EAST by updating its pulsed-power system to compress plasma in two steps by induction fields; high gains of the Lawson trinity parameter and fusion power are both predicted by formulating the HGHF plasma. Both gain rates are faster than the decrease rate of the plasma volume. The formulation is checked by earlier ATC tests. Good agreement between theory and tests indicates that scaling to over 10 T at EAST may be possible by two-step compressions with a compression ratio of the minor radius of up to 3. These results point to a quick new path of fusion plasma study, i.e., simulating the Sun by EAST.

Conceived by Faraday on Aug. 29, 1831, and named by Maxwell, electric induction was described by Lord Rayleigh as “a very big fish indeed not a weed” in a lecture on the hundredth anniversary of Faraday’s birth^1^. Implementation of electric induction into the well-known Maxwell equations with the assumption of displacement current 30 years later unified the apparently disparate fields of light and electromagnetism and changed physics forever^2^,^3^ as the cornerstone of modern physics, astronomy, industry, technology, communication and scientific evaluation of life^3^. As a model of electric induction, the Faraday wheel (FW) not only has been used to explain gamma-ray bursts from the universe^4^ but has also become the dominant engine in electric machines and transformers that power our grid and drive our industries. The creative use of FW by Siemens, Edison and Tesla from 1866 to 1886^5^,^6^ opened a new age of electrification with increasing requirements for clean energy year by year. This increasing energy demand may be terminated within this century thanks to individual and collective efforts globally in building a unique transformer, the International Thermonuclear Experimental Reactor (ITER).

Tokamaks, the most complex and unusual transformers in the world, have been, or are being, implemented at EAST^7^ (Experimental Advanced Superconducting Tokamak, the original HT-7U established by China in 1998), DIII-D, KSTAR, JT60-SA, JET and ITER to study magnetic confinement fusion (MCF) energy^8^,^9^. Because of the inherently pulsed nature of a transformer, RF and NBs (radio frequency and neutral beams) are used to drive the inductively coupled secondary plasma current confined in a vacuum vessel of the tokamak-transformer for steady-state operation; a bootstrap tokamak is formed where major parts of the toroidal current and poloidal field are maintained by plasma self-diffusion with a seeding field^10^,^11^. The ITER 400-s *H-mode* was simulated by EAST over 30 s, providing a foundation for the development of attractive and feasible fusion power plants, such as ITER^7^,^12^ or CFETR (Chinese Fusion Engineering Test Reactor). However, these facilities only consider classical D-shaped plasma in tokamaks for approaching the ignition parameters of plasma and do not incorporate the merits of the Faraday wheel (FW). FW effects are theoretically supported by the linear nature of the Maxwell equations^3^,^4^. Here, a FW with two-step magnetic compressions is suggested for insertion in the existing tokamak to scale up the plasma parameter to a new level, a high-gain high-field (HGHF) state within the plasma, which may facilitate the exploration of fusion energy. Its basic mechanism has been preliminary proven by the Adiabatic Toroidal Compressor (ATC) and TFTR with one-step major-radius magnetic compression, dubbed the Artsimovich-Furth-Ellis axisymmetric magnetic pumping scheme^13^,^14^,^15^,^16^,^17^. The scheme has been collected, formulated and extended here for high-gain plasma accommodated in a conventional D-shaped vacuum vessel, such as in EAST, DIII-D or JET^9^.

## Results

The Lawson criterion is the main target pursued by fusion facilities around the world and specifies a triple product of density *n*, temperature *T* and confinement time 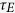 of 3 × 10^21^ keV s m^−3^ for plasma ignition studies^9^. Here, we formulate gain equations for compressed plasma that predict that the HGHF state has high gain not only of the Lawson trinity parameter but also of the fusion power output in the standard vacuum vessel of a tokamak. The tokamak was formerly believed to have significantly larger vacuum vessel peculiarly-shaped as that of the ATC^9^,^13^. Both gain rates of HGHF plasma are much faster than the rate at which the plasma volume decreases. The scaling equations of *L-mode and H-mode* plasmas^9^ both predict the high-gain nature of this highly shifted compressed plasma, and the latter *H-mode* predicts higher gain. The amplified field within the plasma extends its confinement parameter, as checked by ATC tests^13^,^14^,^15^. The ATC results were computed and are listed in Table 1 As expected, Faraday’s lines of force pinch together, resulting in the generation of a high field within the compressed plasma; the field value agrees well with the prediction of the Greenwald density limit for *several energy-confinement times*.

As indicated by both classical *L-mode* scaling^9^ and ITER_98y2_ *H-mode* scaling^18^, the 1972 ATC plasma^13^ might access a special confinement state comparable to the *H-mode* by pure ohm heating, which is inferred from its typical discharge published in 1977^19^. However, enhanced heat transport still exists owing to the limitation of its low poloidal *β*_*p*_. The plasma accessed the state with less than 312 kW of heating power. Calibrating the measured data does indicate an increase in confinement time with compression. With respect to energy-confinement time, the self-generated field due to magnetic compression within the plasma is similar to that of the external field *B*_*t*_; FW effects thus function in tokamak plasma, as mathematically supported by the linear nature of the Maxwell equations^3^.

**Figure 1:**
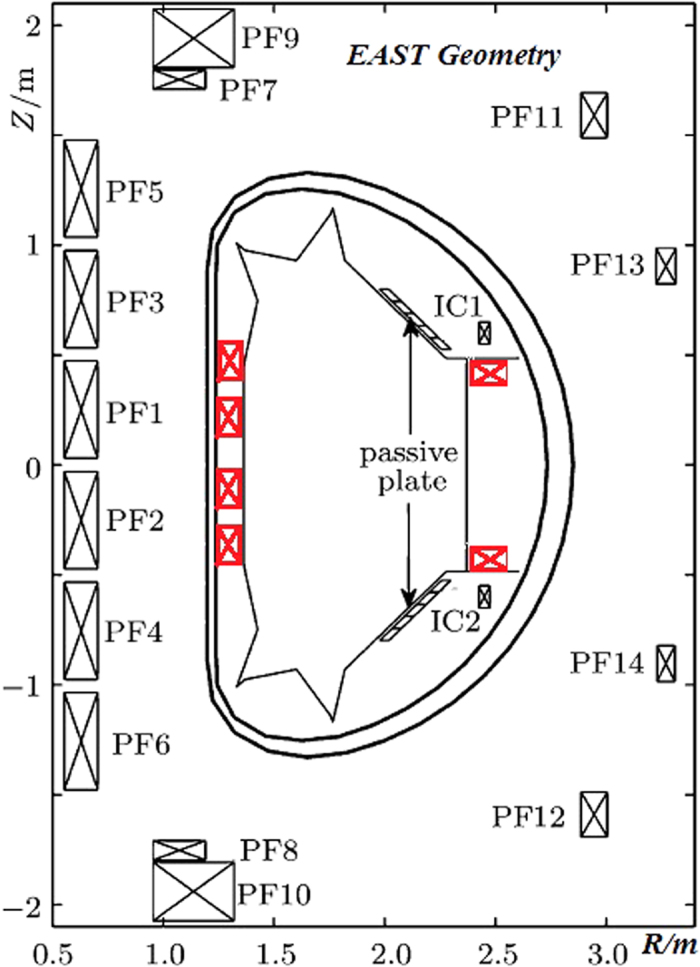
Arrangement of the 4 in-vacuum coils on the left for outboard plasma minor-radius compression and 2 in-vacuum coils on the right for inboard plasma major-radius compression (all new coils are marked in red).

FW effects can be used by EAST to explore the ignition condition by building a time window to amplify the field within the plasma to over 10 T for a super-pulse with a high Lawson parameter. This amplification can be implemented by inserting a new pulse power system in EAST. As shown in Fig. 1, outboard minor-radius compression is first suggested here to synchronously free plasma space of secondary major-radius compression, prepare efficient beam heating and decrease high turbulent transport by increasing poloidal *β*_*p*_^20^. Eddy currents induced in the vacuum chamber of EAST could protect superconducting coils from fast transients of the compression field. The self-amplified high-field is trapped in the plasma and actively controlled by the vertical fields of the servo-control currents in pulse magnets within the vacuum vessel and of the PF coils outside it. High-pressure plasma is thus predicted to be developed by the inductive compression process, in which the Greenwald limit is extended to high density by two-step magnetic compressions in the same vacuum vessel. The new HGHF scheme could contribute more data to the linear scaling law of ITER_98y2_ using the existing tokamak, which is the scientific foundation of ITER and CFETR.

The three scheduled targets of EAST are 1 MA, 10 keV and 1000 s; the first was completed on 28 Nov. 2010, with 1.6 keV and a 1.5-s flat-top phase (shot 34128) in *L-mode*^21^. If plasma shot #34128 was first compressed in its minor radius with a pulsed vertical field at a ratio of 1.5 to 3 to free plasma space of the major-radius compression and decrease high turbulent transport with high poloidal *β*_*p*_, it might have an LH transition. It was subsequently further heated and compressed in the major radius at a ratio of 1.17 to 1.39, identical to that of TFTR^14^. Thus, an extremely HGHF plasma could be realized in EAST according to the following equations and as described in Table 2. The geometric parameters of the compressed plasma are all within the EAST vacuum vessel. The dramatic improvement of EAST using this scheme could not only enable the second goal of 10 keV but also contribute more data to the linear scaling law of ITER_98y2_ at less than 1% of ITER costs. If 1 MA @ 9 keV in EAST is realized by conventional D-shaped plasma within two years, the trinity Lawson parameters in Table 2 could scale by 9/1.6 = 5.6 times at the high-parameter point as pre-compression plasma; EAST will have a trinity Lawson parameter of 3.8 × 10^21^ keV s m^−3^ (*L-mode* case) or 5.2 × 10^21^ keV s m^−3^ (*H-mode* case) and cover the predicted ignition point at 3 × 10^21^ keV s m^−3^.

As to the third goal of 1000 s in EAST, magnetic pumping with pulsed-power technology might be a solution^15^ but would require verification of the net gain of flux in one pumping cycle at non-zero plasma resistance. The plasma flat-top phase could be extended endlessly if the flux loss is fully compensated by the flux gain during one cycle of magnetic pumping, thus achieving the trinity target of EAST for exploring the parameter gain of the Lawson criterion in approaching and covering ignition in the pulse.

## Discussion

### a) Formulas for Compressed Plasma

The equivalent circuit of compressed plasma is an inductor series connected with a resistor, similar to a lossless superconductor circuit while neglecting its resistance. Because the flux in such a circuit is conserved or constant^16^, the equations specifying the conservation of toroidal and poloidal flux as well as plasma entropy are derived as


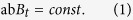


where *B*_*t*_ is the toroidal field (TF) and *a* and *b* are, respectively, the horizontal and vertical minor radius of the plasma ellipse model.

The safety factor is


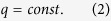


As the temperature and density constraints of the collisional plasma compression,


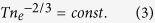



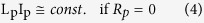


where *L*_*p*_ and *R*_*p*_ are the plasma inductance and resistance in an electric circuit with current *I*_*p*,_ as analyzed by Ejima *et al.* for the tokamak linear-transformer model^22^. *L*_*p*_ = *L*_*i*_ + *L*_*e*_. *L*_*i*_ is the internal inductance of the plasma, which is the integrated partly-current-excited flux linked with it from the plasma axis to its boundary but normalized to *I*_*p*_. *L*_*e*_ is the external inductance linked with all current from the plasma boundary to its magnetic boundary.

After compression, other plasma variables scaling with the major (R) or minor (a) radius are derived as follows^9^,^13^,^14^,^15^,^16^,^17^:


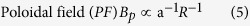



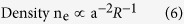



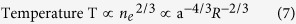



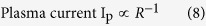



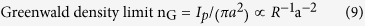


Equation 9 predicts that the threshold of the Greenwald density limit could be linearly extended to a high value by compressing the plasma first in the minor radius to free space and then in the major radius for HGHF. Plasma safety is thus improved with the suggested HGHF method by decreasing disruption risks.


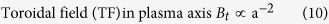



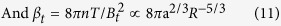



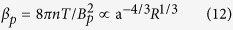


For the first-step outboard minor-radius compression, equation 12 predicts that the poloidal *β*_*p*_ will be enhanced, which will decrease the high turbulent transport and facilitate triggering of H-mode plasma for efficiency heating.

The thermal energy-confinement time is thus derived as


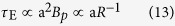


In a beta-limited tokamak as in ref. 9, the gain of fusion power with DT plasma is derived as


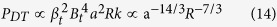


In the same vacuum vessel of fusion plasma, the output power gain of compressed plasma is thus derived as


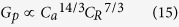


where *C*_*a*_ and *C*_*R*_ are, respectively, the compression ratios of the plasma in the minor radius and major radius by external fields.

Equations 10 and 15 predict the high-gain nature of fusion plasma in the high field in the same vacuum vessel of a tokamak if we compress the plasma first in the minor radius and then in the major radius, i.e., two-step compressions with a beam-heating phase inserted between them. By wasting most of the vacuum vessel volume with compressed plasma, the gains of both the Lawson parameter and fusion power improve significantly; the gain rate is much faster than the rate of volume decrease according to equation 15, and thus it is more efficient and effective to use the existing vacuum vessel volume, not less. The gain equations for compressed plasma are completely formulated here and reasonably account for the gain measurements of ATC and TFTR. For the fusion power gain with the same plasma in a different volume, a smaller minor radius with a high field is much better than a larger minor radius with a low field in the same tokamak. The fusion power gain is predicted to be 48 in ATC and 4.5 in TRTR by equation 15; the gains observed during testing are, respectively, 100 in ATC^23^ and 5.2 in TFTR^14^, slightly better than the predictions.

By combining equations 6, the parameter gain of the Lawson criterion within the compressed plasma is derived as


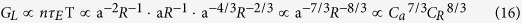


Equation 16 predicts that HGHF plasma also has a high gain in the Lawson criterion by the above magnetic compression; the gain rate is also faster than the rate of volume decrease according to equation 16. These characteristics will facilitate access of the ignition condition by existing tokamak, such as EAST, using the linear nature of the Maxwell equations^3^. The pulse length of discharge for the long-pulse H-modes in EAST is greater than 30 s, which is more than 300 times longer than its energy-confinement time^7^, which we wish to be extended here, together with other two Lawson parameters by magnetic compression. Thus, outboard minor-radius compression should be inserted before ion heating and major-radius compression for high *β*_*p*_ at approximately 0.65R/a = 0.65 × 1.8/0.45 = 2.6 as in ref. 24 to eliminate all penalties of major-radius compression. Plasma can then be heated to the defined temperature, and major-radius compression can begin, as in the ATC.

For practical ohmically heated plasmas in tokamak, the energy-confinement time is empirically scaled as^9^


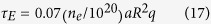


For compression in the major radius, equation 17 can be derived as


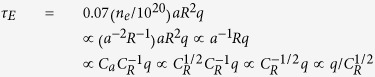


The above derivation clearly demonstrates that the confinement time will decrease with the compression ratio of the major radius but increase slightly with outboard minor-radius compression when *C*_*R*_ < 1, i.e., major-radius expansion. This derivation also reveals that 1/2 power of *C*_*R*_ is in the same position as the beam heating power of the *L-mode* scaling law. *L-mode* was originally analyzed by Goldston, which bears his name^9^. This similarity hints that all heating will shorten the confinement time in a similar manner.

For an ohmically heated tokamak, the saturation limitation of plasma density is empirically scaled as^9^


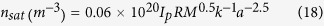


where *I*_*p*_ is the plasma current, *M* is the atomic mass of the ions in amu and *k* is the plasma elongation, *b/a*.

For compressed HGHF plasma in the minor radius, equation 18 can be derived as


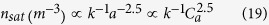


By equation 19, the so-called Improved Ohmic Confinement (IOC) regime could be linearly extended to a high saturation density by the 2.5 power index of the compression ratio in the minor radius.

For external heating with beams of an RF wave and neutral particles, the Goldston *L-mode* scaling law is derived as^9^,


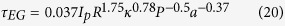


where *P* is the plasma heating power.

The above derivation is further refined as ITER89P in *L-mode* by including JET data^9^ as follows:


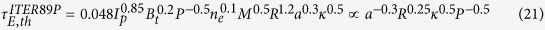


Equation 21 is used in the L-mode scaling of the tokamak. In 1982 at ASDEX, the real *H-mode* was obtained by external heating^24^ and implemented as ITER scaling in 1998, i.e., IPB_98y2_. For IPB_98y2_ scaling, the thermal energy-confinement time is described as^19^


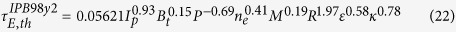


where *ε* is the plasma inverse aspect ratio (a/R).

For compressed HGHF plasma, equations 1, 2, 3, 4, 5, 6, 7, 8, 9, 10, 11, 12 can be inserted into equation 22. For IPB_98y2_ scaling, the thermal energy-confinement time is thus derived as


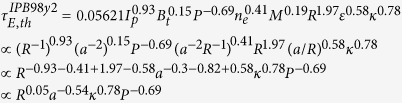



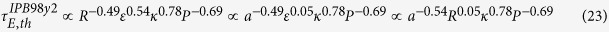


Equation 23 predicts that the energy-confinement time of compressed plasma by external heating could be improved by the 0.54 power index of the compression ratio in the minor radius, together with the 0.78 power index of elongation, as has been purposely explored by the MCF society since 1982.

A degradation of confinement with increased heating power *P* is a natural process and is considered and computed by equations 17, 18, 19, 20, 21 after compression. To decrease the time of energy confinement, all external heating is the same as that for Ohm heating. During compression, the heating power is much less than that of its current drive, as for the ATC case^16^. After compression, the dominant heating power is from the external RF and NBs and not from compression, and only the general Ohm heating remains within the plasma. For pre-compression plasma or post-compression plasma in the steady-state, *P* can be scanned and tuned by external RF and NBs or self-generated fusion alpha particles. An alpha-particle may transfer approximately 90% of its energy to electrons to compensate its 30% losses in compression. If ignition is reached in post-compression, the dominant external heating power is not required, and the remaining heating power left is only for the current drive and power to cure plasma instabilities.

For the same mass of plasma arriving at ignition in the plasma axis, the required heating energy = PΔt and power P in the high-density state of post-compression should be far less than that for pre-compression. Because of the improvement of plasma density and its gradient, post-compression required power is approximately 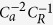 ratio of the pre-compression by simple thermal balance computation without considering the dominant power of the current drive for fueling the decay flux of the plasma inductor in the flattop phase of the plasma pulse. More bootstrap currents could be produced in compressed high-pressure-gradient plasma, which requires less power for the current drive^11^. Thus, a lower non-inductive current fraction is required for post-compression plasma in its flattop phase, and the power of the current drive is reduced. If the scaled-down power multiple of the current drive is assumed to be the ratio of the bootstrap current fraction of the total current in post-compression plasma, it can be simply scaled as^16^


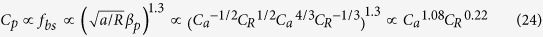


Due to the scaling down of the external heating power, equation 24 predicts that the energy-confinement time of compressed plasma could also be improved by compressed plasma.

In addition, IPB_98y2_ scaling has a negative dependence of *τ*_*E*_ on the plasma beta (β^−0.9^). By eliminating this dependence, as has been evaluated already for some machines, more positive use is found via ref. 25.

### b) Parameter Analysis for Compressed Plasma

For plasma with a circular cross-section, the current distribution is approximated by a simple parabolic model^9^,


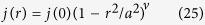


For elongated plasma from above the circular cross-section, the total plasma inductance is thus derived as^9^


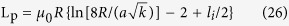


where


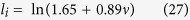


and index


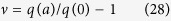


The detailed formulation is provided in ref. 9; *q*(*a*) and *q*(*0*) are safety factors of pre-compression plasma respectively in its boundary and axis. Equation 27 in equation 26 is the normalized internal inductance of the plasma, which is a function of the current distribution within the plasma^9^. The other remains parameters in equation 26 are its external inductance, which must be considered in the compression process because of their natural characteristics of plasma current *I*_*p*_ in space according to Ampere’s law in Maxwell’s equations^16^.

The measured confinement time in the Ohm heating case is computed by^26^


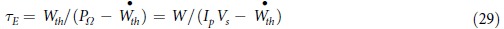


where *V*_*s*_ is the plasma surface voltage and *W*_*th*_ is its thermal storage energy. For the heating power of the ATC case^13^ in 1972, the plasma is compressed and increases in temperature with the increase in thermal energy due to heating only by the inductive current.

**Figure 2:**
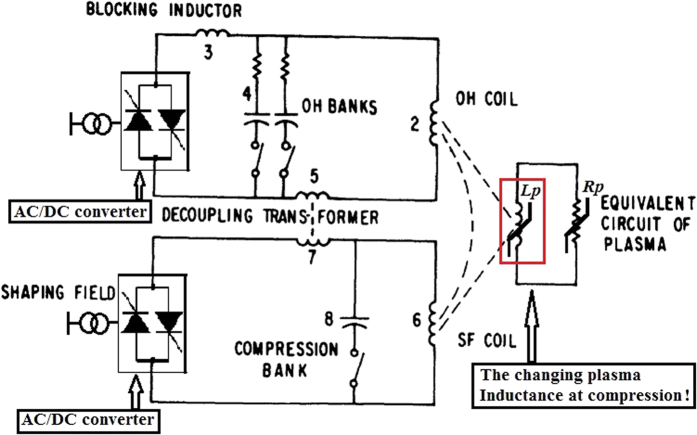
Equivalent circuit of compressed HGHF plasma.

The nonlinear plasma circuit is shown in Fig. 2, which is updated from its typical linear circuit^22^. By Ohm’s law, the circuit equation of the compressed plasma can be simply written as


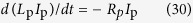


Considering non-zero plasma resistance, equation 27 can be rewritten as


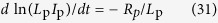


The loss of flux during compression can thus be derived as


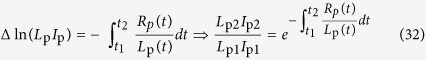


Formula 32 can be used for the value of the natural flux-loss rate of the plasma parameter for CFETR, EAST or ITER. Even without compression, the above equation is still relevant for common D-shaped plasma; tokamak-based devices have a natural Volt-Second (VS) limitation due to their transformer structure. This VS limitation determines the time in the flat-top phases of the plasma discharge after initiating it with approximately 40–50% total transformer flux to overcome the resistive losses of plasma^9^. Here, we observed that VS limitation exists even in the flat-top phases of plasma discharge, which are required to be balanced by a small DC voltage for steady-state operation, as implemented with inductive voltage in EAST shot #41195 for its very long 32-s *H-mode* discharge^7^,^27^,^28^. EAST shot #43336 in ref. 27,28 has a 411-s-long pulse operation, but in *L-mode*, it has a quasi-zero measured loop voltage^27^, i.e., its plasma resistance at discharge is also quasi-zero. By inserting zero resistance in equation 29, ideal constant-flux at compression can be achieved, which may facilitate testing of the above-HGHF scaling law by eliminating flux decay effects^16^. HGHF could also help EAST shot #43336 to be transferred into *H-mode* for simulating the previously described long-plasma-pulse of ITER. Further exploration of the conversion of EAST shot #43336 to *H-mode* by HGHF is warranted and may provide breakthroughs for endlessly long plasma pulses for a CFETR power plant^16^.

### c) ATC in the case of 1972 and 1977

The ATC experimental parameters were sorted^13^,^14^,^15^,^16^ and computed using the equations formulated above and are listed in Table 1. The plasma thermal energy is measured by Thomson scattering of ruby laser light in ATC^13^, as in ref. 29, in which the measured confinement is computed by equation 29. The required information is taken from a plot in ref. 19. Figure 2 of this reference presents the parameter evolution of the ATC tokamak as a function of time, as does the similar discharge in Fig. 1 in ref. 13, except that here the compression time begins at 30 ms and ends at 32 ms, 3 ms earlier than that in ref. 19. The power balance analysis in Figure 4 of ref. 19 incorrectly adds the magnetic energy stored in the plasma internal inductance to the Ohm heating equation, leading to its non-consistent conclusions. Its Ohm heating power is only the product of the plasma current and surface voltage and increases quasi-linearly increased during compression between 33 ms and 34.5 ms. At 33 ms, the Ohm heating power is simply computed as *52 kA x 1.7* *V* = *88.4* *kW.* At 35 ms, the Ohm heating power is *120 kA x 2.6 V* = *312 kW.* From 35 ms, the flux loss could be fully compensated by the transformer flux or RF and NB power to extend its plasma pulse endlessly, as is the case of EAST shot #43336. Unfortunately, neither ATC nor TFTR has performed tests in this manner^13^,^14^,^15^, whereas EAST could further examine the ignition parameter if upgraded with a new pulse power and magnetic system to compress and fuel its decay flux, i.e., plasma current in its flat-top phase. These results suggest that EAST could be rapidly modified to simulate plasmas of ITER or CFETR.

Analysis of the test in Table 1 reveals that the energy gain of plasma during compression scales linearly intermediate with *C*_*R*_ and 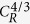; this may be due to the Joule loss of the plasma resistance to its flux, as is described in equation 32.

By equations 4 and 32 with vertical field compression, charging power to the plasma inductor, i.e., the power of the plasma current drive, is derived as


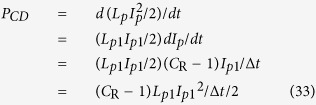


*P*_*CD*_ is calculated as 3.13 MW if inserting ATC parameters, i.e., *L*_*p1*_ = *2.693 μH, I*_*p1*_ = *52 kA*, Δ*t* = *1.5 ms* and *C*_*R*_ = *2.2895* in Table 1 to equation 33. The charging power heats the plasma by Joule loss of the plasma current, i.e., ohm heating. At the beginning of compression, it is simply computed as 52 kA × 1.7 V = 88.4 kW and quasi-linearly increased to 312 kW at 35 ms.

The power of the plasma current drive is approximately 3.13 MW owing to the magnetic compression of ATC. The share of plasma heating is only 0.0884 MW to 0.312 MW, and the heating is naturally absorbed by plasma; the Q value of the ATC plasma inductor load is 10 to 35.4 based on common electrical knowledge. Such heating ionizes more neutrals into the compressed plasma with slightly higher plasma density than predicted by theory in equation 6, as is evident in Table 1. The compression process constitutes a powerful form of current drive, with only slight auxiliary heating. For the ATC case, the maximum heating power at compression is less than 312 kW, but the power of the current drive charging plasma inductor is greater than 3 MW. Compression is only a transition process for compressed plasma with a high Lawson parameter and ends as an equilibrium field of post-compression plasma in its flattop phase; at this phase, we could insert beam power to drive plasma current to obtain a mode of quasi-DC (Direct Current) operation.

The confinement time was improved after compression as confirmed by the ATC tests in 1977. Due to the magnetic compression, the parameter gain of the Lawson criterion is measured at 68.81/2.797 = 24.6 in the plasma center by the well-known Laser-Thomson-Scattering method^13^,^26^. However, the model with ITER_98y2_ predicts a gain at approximately HH_98y2_ × 52.33/2.797 = 1.36 × 18.7 = 25.4, slightly higher than the measured value. The theory predicts that the gain will be nearly identical to the measured value at 35 ms. The gain will subsequently decrease to 8.8 due to the natural decay of the plasma current, which should be fueled by the transformer flux or RF and NB power to extend its plasma pulse in the quasi-DC mode. Quasi *H-mode at HH*_98y2_ = *1.36* did exist in ATC at 35 ms with high current density^13^ at 2–4 MA/m^2^. TFTR produced a negative result for energy-confinement time after compression, possibly due to its nickel influx, current decay and very low current density^30^ at 0.83 MA/m^2^, in which the field of plasma current itself could not suppress the high heat transport to dominate the confinement time, in contrast to ATC.

The electron temperature did not rise adiabatically^13^,^14^, most likely due to the above enhanced turbulent transport driven by the large temperature gradients in both ATC and TFTR, in combination with impurities and synchrotron radiation^19^. Even if the electron temperature is 30% lower than expected, EAST could still arrive at 0.7 × 5.2 × 10^21^ keV s m^−3^  = 3.64 × 10^21^ keV s m^−3^ in *H-mode* case with a minor-radius compression ratio of 3 and weak cover of the ignition point. However, an excessively high electron temperature weakens plasma stability, and *T*_*i*_ > *T*_*e*_ is desirable throughout the compression process. For the core plasma parameters, *T*_*i(0)*_ = *44 keV and T*_*e(0)*_ = *11.5 keV* are achieved at a central electron density of 8.5 × 10^19 ^m^−3^ in TFTR super-shot #76778^31^. Thus, our focus is on the temperature and density of ions that could fuse together for ignition and the generation of power.

### d) Mapping the ITER_98y2_ linear scaling law

What if we incorporate data from ATC in Table 1 to the Map of ITER_98y2_ scaling law? Before compression, ATC is in *L-mode* and under the line. After compression at 35 ms, its confinement time is comparable to the *H-mode* and above the line at *HH*_*98y2*_ = *1.36*. If JET is further implemented with HGHF plasma by two-step compressions as suggested for EAST in Table 2, it might fill the blank space between JET and ITER in ITER_98y2_ scaling. Linear scaling of ITER_98y2_ may exist at large as predicted in ref. 9 due to the linear nature of the Maxwell equations^3^.

Forty-two years ago, *Nature* published an article titled “Fusion power changes gear” by its Washington correspondent describing ATC results^32^; however, fusion is historically geared to the diverter-embedded D-shaped plasma in emerging DIII-D, EAST, KSTAR, JET, JT60-SA and ITER^9^. As mathematically evident in equation 22, the confinement time is extended by the 0.78 power index of elongation. As further indicated by equation 23, the formulated −0.54 index in HGHF gives us confidence in the power of minor radius to extend the confinement time and approach the ignition parameter, as suggested in table 2, as a new path for compressed plasma for EAST. If the Greenwald density limit and confinement time in EAST are extended and measured as predictions of equations 9 and 22, a fast track for fusion power may emerge by dotting more points on the linear scaling line of ITER_98y2_, finally resulting in the predicted change in gear in 2015.

## Methods

### Step-by-step Compressions for HGHF plasma

Pre-compression plasma is first established to accommodate the flux-seed of the toroidal field in the existing tokamak upgraded with a magnetic compression system in the minor and major radii. The trapped flux in plasma is first compressed at the minor radius by moving it to the low field side of the vacuum vessel with maximum major radius to free compression space of the secondary major-radius compression. The compression can be achieved by dramatically increasing the upgraded inner vertical field and controlling the outer vertical field as a fixed toroidal field, identical to that of ATC but in the opposite outboard direction to shape the plasma with right-predicted elongation. In this stage, the plasma has high density for efficiently absorbing more beam power due to its high opacity, similar to that of DIII-D^33^. The plasma is subsequently compressed in the major radius for HGHF plasma similar to ATC and TFTR^13^,^14^. The amplified ion and beam energy could allow EAST to approach the ignition parameter as computed in Table 2. The testing results for ATC and TFTR are encouraging because the theoretical model agrees well with the experiment results, as analyzed in Table 1.

In closing, it may be interesting to review the predictions of Furth in 1990^34^. The site of ITER has moved from central Europe to southern France. ITER also eliminates the function of prototypical electric power generation. The timing of its DT operating phase has been postponed from 2005 to approximately 2033, nearly 30 years deferred. The FW conversion of the tokamak and confinement improvement at ATC could help accelerate the progression of magnetic confinement fusion at HGHF for EAST or other existing MCF facilities^9^.

## Additional Information

**How to cite this article**: Li, G. High-Gain High-Field Fusion Plasma. *Sci. Rep.* **5**, 15790; doi: 10.1038/srep15790 (2015).

## Figures and Tables

**Table 1:** ATC plasma parameters sampled at three times during discharge, *C*_*R*_ = 2.289/*C*_*a*_ = 1.513 (1.57 by the theory model).

Time (ms)	25	35	45
Minor radius (*a/m*)^19^	*0.165*	*0.105*	*0.105*
Major radius (*R/m*)^19^	*0.87*	*0.38*	*0.38*
Density (*n*_*e*_*/m*^−*3*^)	*2.3* × *10*^*19*^	*16* × *10*^*19*^ *(Theory 1.357* × *10*^*20*^)	*10* × *10*^*19*^
Density (<*n*_*e*_>*/m*^−*3*^)	*1.0* × *10*^*19*^	*5.8x10*^*19*^ *(Theory 5.634* × *10*^*19*^)	*3.5* × *10*^*19*^
Temperature (*T*_*e0*_*/keV*)	*0.95*	*2.35* (2.86 by equation 4)	1.6
Temperature (<*T*_*e*_>*/keV*)	*0.26*	*0.63* (0.78 by equation 4)	*0.39*
Temperature (*T*_*i0*_*/keV*)^13^	0.2	0.6	N/A
Toroidal field at plasma axis *B*_*t*_ (*R*)/T	*1.5*	*3.4*	*3.4*
Plasma current (*I*_*p*_*/kA*), Voltage (*V*_*s*_ */V*)	60, 2.6	120, 2.6	70, 1.7
Plasma internal inductance (*L*_*i*_*/μH*)	0.7885	0.3444	0.3444
Plasma inductance (*L*_*p*_*/μH*)	2.693	0.9965 (1.176 by equation 4)	0.9965 (1.176 by equation 4)
Plasma resistance (*R*_*p*_*/μΩ*)	43.3	*21.7*	*24.7*
Time const. of plasma decay (*L*_*p*_*/R*_*p*_)	62.2 ms	45.9 ms	40.3 ms
Plasma poloidal beta (*β*_*p*_)	*0.32*	*0.36 (Theory 0.42)*	*0.48*
Total magnetic energy (*W*_*m*_*/kJ*)	1.499	2.621	0.892
Total plasma energy (*W*_*p*_*/kJ*)	0.235	0.561	N/A
Aspect ratio (*R/a*)	*5.273*	*3.619*	*3.619*
*τ*_*Ee*_ (Theory/ms) by equation 17	*3.41*	*2.52*	2.6
*τ*_*Ee*_ (Measure/ms)	*1.28 (L-mode)*	*1.83 (?H-mode)*	*1.54 (?H-mode)*
*τ*_*E*_ (ITER_98y2_/ms) by equation 22	N/A	1.3483	1.2897
HH_98y2_	N/A	*1. 36*	1.19
Lawson parameter of plasma center/keV s m^−3^	2.797 × 10^16^	6.881 × 10^17^ (5.233 × 10^17^ by theory)	2.464 × 10^17^
Saturation density (*n*_*e*_*/m*^−*3*^)	*7* × *10*^*19*^	*3.46* × *10*^*20*^	*2.02* × *10*^*20*^

Some data are taken from ref. 13 and ref. 19 for model calibration.

**Table 2:** Scaling Parameters of EAST by two-step compressions using shot #34128 as pre-compression plasma.

Parameter	EAST Parameters if *C*_*a*_ = *1.5&C*_*R*_ = *1.17*	EAST Parameters if *C*_*a*_ = *3&C*_*R*_ = *1.39*
Minor radius (*a*)	*a* = *0.45* → *0.3 m*	*a* = *0.45* → *0.15 m*
Major radius (*R*)	*R* = *1.65*–*1.93 m*	*R* = *1.5*–*2.1 m*
First minor-radius compression	*R/a* = *1.93 m/0.32 m*	*R/a* = *2.08 m/0.17 m*
Final major-radius compression	*R/a* = *1.65 m/0.3 m*	*R/a* = *1.5 m/0.15 m*
Density (*n*)	*4.74* × *10*^*19*^ *m*^−*3*^	*2.252* × *10*^20^ *m*^−*3*^
Temperature (*T*)	*1.91* × *1.6* = *3.05* *keV*	*5.389* × *1.6* = *8.62* *keV*
TF field at plasma axis *B*_*t*_(*R*)	*6.5* *T*	*30.87* *T*
Plasma current (*I*_*p*_)	*1. 09* *MA*	*1.2* *MA*
Gain of plasma energy (*W*_*p*_)	*C*_*a*_^*4/3*^ *C*_*R*_^*4/3*^ = *1.717* × *1.233* = *2.116*	*C*_*a*_^*4/3*^ *C*_*R*_^*4/3*^ = *4.3267* × *1.551*=*6.712*
Aspect ratio after final compression (*R/a*)	*6.0* → *5.5*	*12.2* → *10*
*τ*_*E*_(ITER89-P, ITER98y2/ms)	*158.1(L-mode)* → *227.6(H-mode)*	*349.8 (L-mode)* → *486.5 (H-mode)*
Gain of Lawson trinity parameter	*2.5756* × *1.52* = *3.915*	*12.98* × *2.4* = *31.24*
Lawson trinity parameter at the plasma axis	*2.29* × *10*^*19*^ *(L-mode)* → *3.29* × *10*^*19*^ *(H-mode)*	*0.679* × *10*^*21*^ *(L-mode)* → *0.944* × *10*^*21*^ *(H-mode)*
Gain of fusion power	*C*_*a*_^*14/3*^ *C*_*R*_^*7/3*^ = *9.57*	*C*_*a*_^*14/3*^ *C*_*R*_^*7/3*^ = *363.3*

*R* = *1.8* *m, a* = *0.45 m, n*_*e*_ = *1.8* × *10*^*19 *^*m*^−*3*^*, B*_*T*_ = *2.45* *T, T*_*e*_ = *1.6 keV, Plasma Current I*_*p*_ = *1 MA* (EAST shot #34128).
